# 

**Published:** 1905-12-30

**Authors:** 


### DEATH.

**Notices of Births, Deaths, and Marriages** are inserted in this column at a charge of 2s. 6d. for an announcement not exceeding four lines.

On December 11, at 16 The Mount, York (residence of her sister, Dr. Fraser), JEANNIE FRASER, daughter of Hugh I. Fraser, of Inverness, N.B., formerly nurse at St. Thomas's Hospital, London. (3255)

### NOTICE TO CORRESPONDENTS.

All MSS., Letters, Books for Review, and other matters intended for the Editor should be addressed The Editor, "The Hospital," The Hospital Buildings, 28 & 29 Southampton Street, Strand, W.C.

The Editor cannot undertake to return rejected MS., even when accompanied by stamped directed envelope.

**Notice to Advertisers and Subscribers.**

All Advertisements, Orders for Copies of The Hospital, and Business Communications should be addressed to **THE MANAGER (Not to the Editor),** The Hospital, 28 & 29 Southampton Street, Strand London, W.C.

The Cost of Subscription, post free, is at follows:—Per week, 3½d.; per quarter, 3s. 3d.; per half-year, 6s. 6d.; per annum, 13s. Foreign and Colonial:—Per annum, 19s. 6d., payable, in all cases, in advance.

**NOTICE.—Vol. XXXVIII. of "The Hospital" April to September 1905), in handsome cloth binding, will shortly be on sale at the office of this Journal, price 10s. 6d. Cases for binding the Numbers contained in this Volume 2s. Index to Vol. XXXVIII., 3½d., post free.**

**The Monthly Part for December is now ready. Price 1s., by post, 1s. 3d.**

### BOOKS RECEIVED.

Scientific Press, Ltd.

"Notes on General Practice." By S. M. Hebblethwaite, M.D.

J. B. Lippincott Co.

"Krehl's Clinical Pathology." Translated by A. W. Hewlett, M.D.

"Therapeutics, its Principles and Practice." By H. C. Wood, M.D.

"International Clinics." Fifteenth series. Vol. III.

T. Fisher Unwin.

"Nature and Origin of Living Matter." By H. Charlton Bastian, M.A.

Rebman, Ltd.

"Clinical Obstetrics." By R. Jardine, M.D., F.R.S. Edin.

### Medical Appointments and Vacancies.

### VACANCIES.

Birmingham, University of.—Demonstratorship in Anatomy.

Bristol Royal Infirmary.—Pathologist, Bacteriologist, and Director of Clinical Laboratories.

Bristol, University College, Faculty of Medicine.—Professor of Pathology.

Chelsea Hospital for Women, Fulham Road, S.W.—Resident Medical Officer.

East London Hospital for Children and Dispensary for Women, Shadwell, E.—House Physician.

Hartshill, Stoke-upon-Trent, North Staffordshire Infirmary and Eye Hospital.—Junior House Surgeon.

Hastings, St. Leonards, and East Sussex Hospital.—Assistant House Surgeon.

London Fever Hospital, Liverpool Road, N.—Resident Medical Officer.

National Hospital for the Paralysed and Epileptic, Queen Square, Bloomsbury, W.C.—Resident Medical Officer.

Northampton General Hospital.—Honorary Assistant Surgeon.

Ryde, Royal Isle of Wight County Hospital.—Resident House Surgeon.

Samaritan Free Hospital for Women, Marylebone Road, N.W.—Clinical Assistants.

Seamen's Hospital Society, Greenwich.—Two Assistant Physicians and one Assistant Physician or Surgeon for Diseases of the Throat, Nose, and Ears. Also Pathologist.

Southport Infirmary.—Resident Junior House and Visiting Surgeon, unmarried.

Stafford, Staffordshire General Infirmary.—Assistant House Surgeon.

Tiverton, Devonshire, Infirmary and Dispensary.—House Surgeon and Dispenser.

Wolverhampton and Staffordshire General Hospital.—Assistant House Physician.

### Medical Appointments.

### IMPORTANT NOTICE.

A Useful Xmas Present for Nurses.

THE

### HOSPITAL

### Calendar & Notebook

FOR

### 1906.

**This compact and useful little Notebook, which is of a convenient size for the pocket, is in appearance an exact reproduction of THE HOSPITAL Journal in miniature and contains much information of value to nurses, including: Hints for nurses; How to advertise; How to reply to an advertisement; Hints on how to accept a post; Measures; Apothecaries' and other weights, &c., &c.**

A Useful Xmas Present for Nurses.

### To Nurses.

THE HOSPITAL Calendar and Notebook will be forwarded **Post Free** on receipt of **Three Halfpenny Stamps.** Nurses requiring additional Copies can obtain One Dozen, post free, for Six Penny Stamps.

**BINDING CASES,** in New Pegamoid Leather Cloth, gilt lettered, specially designed to take **THE HOSPITAL Calendar and Notebook,** with loop for hanging from Wallet or Chatelaine with One Copy of the Calendar, **3½d.** post free. These Cases are strongly made, and can be used from year to year, as they do not contain the year nor any lettering other than the title of the Calendar.

The Diary will shortly be ready, and it is advisable to make immediate application to avoid disappointment, as the edition is limited and no reprints will be issued when it is exhausted.

*Address*—The Manager, Calendar Dept., THE HOSPITAL,
28 & 29 Southampton Street, Strand, London, W.C.

